# Contribution of a lectin, LecM, to the quorum sensing signalling pathway of *Ralstonia solanacearum* strain OE1‐1

**DOI:** 10.1111/mpp.12757

**Published:** 2018-11-06

**Authors:** Kazusa Hayashi, Kenji Kai, Yuka Mori, Shiho Ishikawa, Yumeto Ujita, Kouhei Ohnishi, Akinori Kiba, Yasufumi Hikichi

**Affiliations:** ^1^ Laboratory of Plant Pathology and Biotechnology Kochi University Nankoku Kochi 783‐8502 Japan; ^2^ Graduate School of Life and Environmental Sciences Osaka Prefecture University Sakai Osaka 599‐8531 Japan; ^3^ Research Institute of Molecular Genetics, Kochi University Nankoku Kochi 783‐8502 Japan

**Keywords:** LecM, quorum sensing, *Ralstonia solanacearum*

## Abstract

The soil‐borne bacterium *Ralstonia solanacearum* invades the roots and colonizes the intercellular spaces and then the xylem. The expression of *lecM*, encoding a lectin LecM, is induced by an OmpR family response regulator HrpG in *R. solanacearum* strain OE1‐1. LecM contributes to the attachment of strain OE1‐1 to the host cells of intercellular spaces. OE1‐1 produces methyl 3‐hydroxymyristate (3‐OH MAME) through a methyltransferase (PhcB) and extracellularly secretes the chemical as a quorum sensing (QS) signal, which activates QS. The expression of *lecM* is also induced by the PhcA virulence regulator functioning through QS, and the resulting LecM is implicated in the QS‐dependent production of major exopolysaccharide EPS I and the aggregation of OE1‐1 cells. To investigate the function of LecM in QS, we analysed the transcriptome of *R. solanacearum* strains generated by RNA sequencing technology. In the *lecM* mutant, the expression of positively QS‐regulated genes and negatively QS‐regulated genes was down‐regulated (by >90%) and up‐regulated (by ~60%), respectively. However, *phcB* and *phcA *in the *lecM* mutant were expressed at levels similar to those in strain OE1‐1. The *lecM* mutant produced significantly less ralfuranone and exhibited a significantly greater swimming motility, which were positively and negatively regulated by QS, respectively. In addition, the extracellular 3‐OH MAME content of the *lecM* mutant was significantly lower than that of OE1‐1. The application of 3‐OH MAME more strongly increased EPS I production in the *phcB*‐deleted mutant and strain OE1‐1 than in the *lecM* mutant. Thus, the QS‐dependent production of LecM contributes to the QS signalling pathway.

## Introduction

Many bacteria regulate their cooperative activities and physiological processes through quorum sensing (QS), in which bacterial cells communicate with each other by releasing, sensing and responding to small diffusible signal molecules, called QS signals (Ham, 2013). QS signals accumulate in the environment as the density of the bacterial population increases, and the bacteria monitor this information to track changes in their cell numbers. QS controls the expression of genes involved in activities that are beneficial when performed by groups of bacteria acting in synchrony (Rutherford and Bassler, 2012). Importantly, many pathogenic bacteria use cell–cell signalling to regulate the expression of factors that contribute to virulence (Ham, 2013).

Bacterial wilt caused by the soil‐borne, plant‐pathogenic, Gram‐negative bacterium *Ralstonia solanacearum *is a devastating bacterial plant disease in tropical, subtropical and warm‐temperature regions worldwide (Mansfield *et al*., 2012). *Ralstonia* *solanacearum *normally invades plant roots from the soil through wounds or via natural openings from which secondary roots emerge (Araud‐Razou *et al*., 1998), and colonizes the intercellular spaces of the root cortex and vascular parenchyma (Hikichi, 2016; Vasse *et al*., 1995). After invasion into the intercellular spaces, cells of *R. solanacearum* strain OE1‐1 attach to the surfaces of host plant cells (Hikichi *et al*., 2017). *Ralstonia solanacearum *produces a lectin, LecM (RS‐IIL), which is encoded by *lecM* and exhibits mannose, fructose, fucose, galactose and arabinose affinities (Sudakevitz *et al*., 2002, 2004). Meng *et al*. (2015) reported that *R. solanacearum* strain UW551 at cooler temperatures up‐regulates the expression of *lecM*, which is required for its full virulence, and LecM functions inside the plant, not only during attachment to root surfaces. *lecM* expression is positively regulated by a transcriptional regulator of the *hrp* regulon, HrpG (Mori *et al*., 2016; Valls *et al*., 2006), and LecM is involved in the attachment of *R. solanacearum* cells to the surfaces of plant cells after invasion into intercellular spaces (Mori *et al*., 2016). OE1‐1 then expresses *hrp* genes, constructs the type III secretion machinery and translocates effectors into host cells (Hikichi *et al*., 2017), inducing the production of the phosphatidic acid phosphatase of host plants, which dephosphorylates phosphatidic acid into diacylglycerol in the choroloplast membranes of the host plant (Nakano *et al*., 2013). This leads to the suppression of jasmonic acid‐ and reactive oxygen‐mediated defences, which are induced through Sec14P‐mediated phospholipid signalling to produce phosphatidic acid in chloroplast membranes (Kiba *et al*., 2014; Nakano *et al*., 2013). This allows OE1‐1 to escape the innate immunity of host plants and to vigorously multiply on host plant cells (Hikichi *et al*., 2017).

The vigorous growth of *R. solanacearum* after escaping the innate immunity of host plants leads to QS (*phc* QS). *Ralstonia solanacearum *strains AW1 and K60 produce methyl 3‐hydroxypalmitate as a QS signal (Flavier *et al*., 1997; Kai *et al*., 2015). In addition, *R. solanacearum *strains OE1‐1 and GMI1000 produce methyl 3‐hydroxymyristate (3‐OH MAME) as a QS signal (Kai *et al*., 2015). These QS signals are synthesized by the methyltransferase PhcB (Fig. S1, see Supporting Information; Flavier *et al*., 1997; Kai *et al*., 2015). When the QS signals reach a threshold level, they reduce the ability of the histidine kinase PhcS to phosphorylate the response regulator PhcR, resulting in elevated levels of functional PhcA, an LysR‐type transcriptional regulator, which plays a central role as a global virulence regulator in *phc *QS (Genin and Denny, 2012; Hikichi *et al*., 2017).

*Ralstonia solanacearum* synthesizes aryl‐furanone secondary metabolites, known as ralfuranones A, B, I, J, K and L, which are secreted extracellularly (Kai *et al*., 2016, 2014; Pauly *et al*., 2013). Ralfuranone I is a precursor for other ralfuranones. The expression of ralfuranone synthase, encoded by *ralA*, is dependent on PhcA functioning through *phc* QS and is involved in the biosynthesis of ralfuranones (Kai *et al*., 2016, 2014; Schneider *et al*., 2009; Wackler *et al*., 2011). Interestingly, ralfuranones are implicated in a positive feedback loop in *phc* QS (Fig. S1; Hikichi *et al*., 2017; Mori *et al*., 2018b).

The production of major exopolysaccharide EPS I, which is required for *R. solanacearum* virulence, is positively regulated by *phc* QS (Huang and Schell, 1995). The *epsB* gene is included in the *eps* operon, which is involved in EPS I biosynthesis, and its expression is induced by PhcA functioning through *phc* QS. LecM production is also induced by PhcA functioning through *phc* QS (Meng *et al*., 2015; Mori *et al*., 2016) and is involved in the production of *phc* QS‐dependent major exopolysaccharide EPS I, which leads to the aggregation of OE1‐1 cells (Fig. S1; Mori *et al*., 2016). In addition, a comparison of the levels of gene expression of wild‐type (WT) *R. solanacearum *strain GMI1000 and the *phcA*‐deleted mutant during tomato colonization revealed that PhcA positively regulates *lecM* expression in strain GMI1000, which infects xylem vessels of tomato (Khokhani *et al*., 2017).

In this study, we first assayed the expression of *epsB *in the* lecM* mutant (OE1‐1‐*lecM*::EZ Tn*5*; Mori *et al*., 2016). To elucidate the function of LecM in *phc* QS, we examined the transcriptome profile of the* lecM* mutant compared with the *phc* QS‐deficient mutants, as well as the wild‐type (WT) OE1‐1 strain (Kanda *et al*., 2003), using RNA sequencing (RNA‐seq) technology. We then analysed the involvement of LecM in *phc* QS‐dependent virulence‐related phenotypes.

## Results

### The *lecM* mutation leads to significantly reduced *epsB* expression

The *lecM* mutant produces significantly less EPS I than does the parent strain OE1‐1 and the native *lecM*‐expressing complemented *lecM* mutant (*lecM*‐comp; Mori *et al*., 2016). Incubation on Hara–Ono medium (Hara and Ono, 1983) for 48 h allowed us to visibly observe EPS production by *R. solanacearum* strain OE1‐1, the *lecM* mutant and *lecM*‐comp (Fig. S2, see Supporting Information). The *lecM* mutant (OE1‐1‐*lecM*::EZ Tn*5*; Mori *et al*., 2016) exhibited significantly reduced EPS production on Hara–Ono medium. EPS production by the native *lecM*‐expressing complemented *lecM* mutant (*lecM*‐comp; Mori *et al*., 2016) was similar to that of strain OE1‐1.

**Figure 1:**
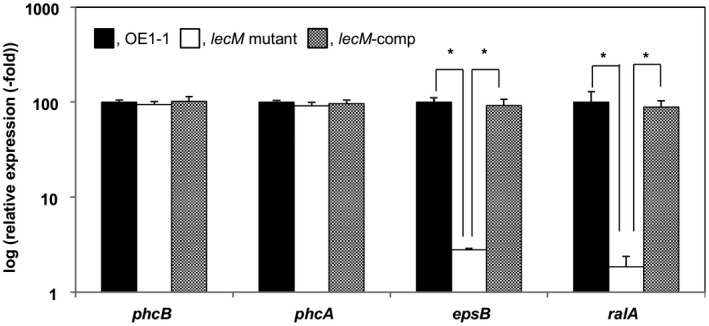
Influence of the *lecM* mutation on the expression of *phc* quorum sensing (QS)‐related genes *phcB* and *phcA*, and the positively *phc* QS‐mediated virulence genes *epsB* and *ralA*, of *Ralstonia solanacearum*. *Ralstonia solanacearum* OE1‐1, *lecM* mutant (OE1‐1‐*lecM*::EZ Tn*5*) and native *lecM*‐expressing complemented *lecM* mutant (*lecM*‐comp) strains were grown in ¼ × M63 medium [to an optical density at 600 nm (OD_600_) = 0.3]. Total RNA was then extracted from the bacterial cells. The *rpoD* gene was used as an internal control for quantitative reverse transcription‐polymerase chain reaction. The gene expression levels are presented relative to the *rpoD* expression level. The experiment was conducted at least twice using independent samples, with similar results. Results for a single representative sample are provided. Values are presented as the means ± standard deviations of three replicates. Asterisks indicate values significantly different from those of *lecM* mutant cells (*P *< 0.05, *t*‐test).

We then assessed the expression of *epsB* in *R. solanacearum* strains grown in ¼ × M63 medium using quantitative reverse transcription‐polymerase chain reaction (qRT‐PCR) assays. The *epsB* expression level was significantly lower in the *lecM* mutant than in OE1‐1 or *lecM*‐comp (*P* < 0.05, *t‐*test, Fig. 1).

### The *lecM* mutation affects the gene expression regulated by 3‐OH MAME perception

**Figure 2:**
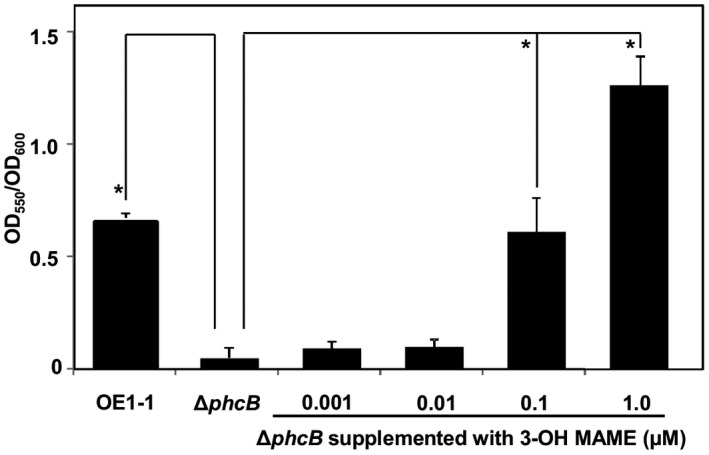
Cell aggregation by *Ralstonia solanacearum* OE1‐1, the *phcB*‐deleted mutant (Δ*phcB*) and Δ*phcB* supplemented with methyl 3‐hydroxymyristate (3‐OH MAME). The OE1‐1 and Δ*phcB* cells were incubated in ¼ × M63 medium in the wells of polyvinylchloride microtitre plates. Δ*phcB* cells were also incubated in ¼ × M63 medium supplemented with 3‐OH MAME at concentrations of 0.001–1.0 µm. The wells were stained with crystal violet. The experiment was repeated three times, with seven technical replicates in each experiment. Asterisks indicate values significantly different from those of strain OE1‐1 (*P* < 0.05, *t*‐test).

Supplementation with 3‐OH MAME leads to the recovery of EPS and ralfuranone productivity in Δ*phcB* (Kai *et al*., 2015). To determine the concentration of 3‐OH MAME for supplementation to Δ*phcB*, we assessed the *phc* QS‐dependent cell aggregation of Δ*phcB* supplemented with 3‐OH MAME at concentrations of 0.001–1.0 µm. The aggregation level of Δ*phcB* cells increased significantly as the 3‐OH MAME concentration increased from 0.1 to 1.0 µm (*P* < 0.05, *t*‐test, Fig. 2).

We performed transcriptome analysis using RNA‐seq of *R. solanacearum* strains OE1‐1 and the *lecM* mutant, but also Δ*phcB*, Δ*phcB* supplemented with 1.0 µm 3‐OH MAME and the *phcA*‐deleted mutant (Δ*phcA*). For transcriptome analyses using RNA‐seq, total RNA was isolated from cells of *R. solanacearum* strains OE1‐1, the *lecM* mutant, Δ*phcB*, Δ*phcB* supplemented with 1.0 µm 3‐OH MAME and Δ*phcA*, and cultured in ¼ × M63 medium [to an optical density at 600 nm (OD_600_) = 0.3]. Cytoplasmic ribosomal RNA was removed from total RNA, resulting in a final RNA yield of 400 ng for each sample. The isolated RNA was subjected to Illumina RNA‐seq. The RNA samples were fragmented and ligated with adaptors prior to cDNA synthesis and PCR amplifications. Two biologically independent experiments were conducted for each strain. We obtained 41.8 and 46.0, 45.6 and 44.0, 46.5 and 43.9, 44.0 and 47.3, and 45.3 and 44.4 million 100‐bp paired‐end reads from strains OE1‐1, the *lecM* mutant, Δ*phcB*, Δ*phcB* supplemented with 1.0 µm 3‐OH MAME and Δ*phcA*, respectively. By iterative alignment, 41.0 and 45.0, 45.1 and 42.5, 42.5 and 43.5, 42.5 and 45.8, and 41.4 and 43.0 million 100‐bp paired‐end reads, respectively, were successfully mapped to the* R. solanacearum* strain GMI1000 reference genome (Salanoubat *et al*., 2002). The mapping of the OE1‐1 RNA‐seq reads to the GMI1000 genome resulted in the identification of 4491 protein‐coding transcripts.

The normalized gene expression levels for *R. solanacearum* strain OE1‐1 and the other strains were compared to detect differentially expressed transcripts. The read counts obtained for each sample were expressed as the fragments per kilobase of open reading frame per million fragments mapped (FPKM) normalized prior to analysis for differentially expressed genes. Genes were considered to be differentially expressed if they exhibited log_2_(fold changes) of ≥1 or ≤−1 (fold changes of ≥2 or ≤−2).

**Figure 3:**
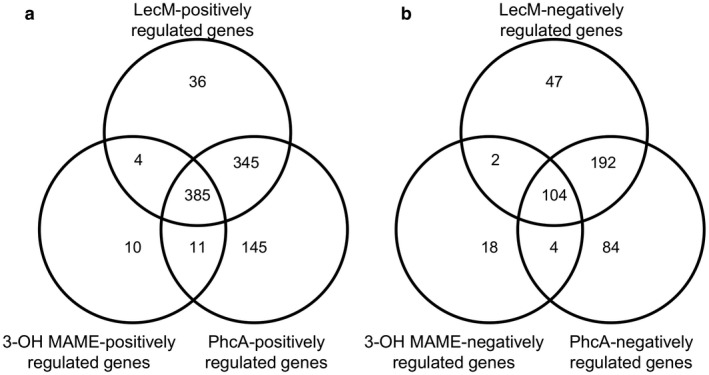
Number of genes for which expression was regulated by methyl 3‐hydroxymyristate (3‐OH MAME), PhcA and LecM encoded in *lecM* in *Ralstonia solanacearum* strain OE1‐1. The transcriptome analyses using RNA sequencing (RNA‐seq) were performed on RNA from the *R. solanacearum* *phcB*‐deleted mutant (Δ*phcB*), Δ*phcB* supplemented with 1.0 µm methyl 3‐hydroxymyristate (3‐OH MAME), *phcA*‐deleted mutant (Δ*phcA*) and *lecM* mutant (OE1‐1‐*lecM*::EZ Tn*5*). Numbers of genes that exhibited expression level log_2_(fold changes) of ≤−1 (a) or ≥1 (b) in Δ*phcB* supplemented with 1.0 µm 3‐OH MAME relative to the expression levels in Δ*phcB*, and expression level log_2_(fold changes) of ≤−1 (a) or ≥1 (b) in Δ*phcA *and *lecM *mutant relative to the expression levels of strain OE1‐1. The fragments per kilobase of open reading frame per million fragments mapped (FPKM) values of OE1‐1, Δ*phcB*, Δ*phcA* and *lecM* mutant strains were normalized prior to the analysis of differentially expressed genes.

We detected 744 genes that were expressed at significantly lower levels in Δ*phcB* than in OE1‐1 (Table S1, see Supporting Information). The application of 3‐OH MAME led to significantly enhanced expression levels of 410 of these genes, suggesting that their expression is positively regulated by 3‐OH MAME perception (3‐OH MAME‐positively regulated genes) (Fig. 3a). Among the 886 genes (PhcA‐positively regulated genes) that were expressed at significantly lower levels in Δ*phcA* than in strain OE1‐1, 396 genes were 3‐OH MAME‐positively regulated genes, suggesting that their expression is positively regulated by *phc* QS (*phc* QS‐positively regulated genes). Among the PhcA‐positively regulated genes, more than 400 genes were not 3‐OH MAME‐positively regulated genes, suggesting that positive regulation by PhcA may be independent of 3‐OH MAME production. We also detected 770 genes (LecM‐positively regulated genes) that were expressed at significantly lower levels in the *lecM* mutant than in strain OE1‐1. Among the LecM‐positively regulated genes, 385 genes, including the major EPS (i.e. EPS I) production‐related genes, such as those in the *eps* operon (i.e. *epsR* and *xpsR*), the type VI secretion system‐related genes, plant cell wall degradation enzyme genes (i.e. *pme* and *egl*), two‐component system‐related genes (i.e. *solI* and *solR*) and some effector genes secreted through the type III secretion systems (i.e. *RSc1723* and *Rsp0323*, *ripO1*), were included in the *phc* QS‐positively regulated genes (Fig. 3a; Table S2, see Supporting Information). In addition, 345 LecM‐positively regulated genes were also positively regulated by PhcA, but not by 3‐OH MAME (Fig. 3a).

We also detected 348 genes that were expressed at higher levels in Δ*phcB* than in OE1‐1 (Table S1). Among them, 3‐OH MAME application increased the expression levels of 128 genes (3‐OH MAME‐negatively regulated genes) in Δ*phcB* (Fig. 3b)*.* Among the 384 genes (PhcA‐negatively regulated genes) that were expressed at significantly greater levels in Δ*phcA* than in strain OE1‐1, 108 genes were 3‐OH MAME‐negatively regulated genes, suggesting that their expression is negatively regulated by *phc* QS (*phc* QS‐negatively regulated genes). However, among the PhcA‐negatively regulated genes, more than 250 genes were not 3‐OH MAME‐negatively regulated genes, suggesting that the negative regulation by PhcA may be independent of 3‐OH MAME production. The *lecM* mutation resulted in the significantly reduced expression of 345 genes (LecM‐negatively regulated genes). Among these, 104 genes, including flagellar motility‐related genes, such as *fliC*, type III secretion‐related genes and chemotaxis‐related genes, were *phc* QS‐negatively regulated genes (Fig. 3b; Table S3, see Supporting Information). In addition, 192 genes were negatively regulated by PhcA, but not by 3‐OH MAME (Fig. 3b).

The expression levels of all transcripts in the *lecM* mutant were positively correlated with those in Δ*phcB* and Δ*phcA* (Fig. S3, see Supporting Information).

### The *lecM* mutation leads to reduced ralfuranone production

**Figure 4:**
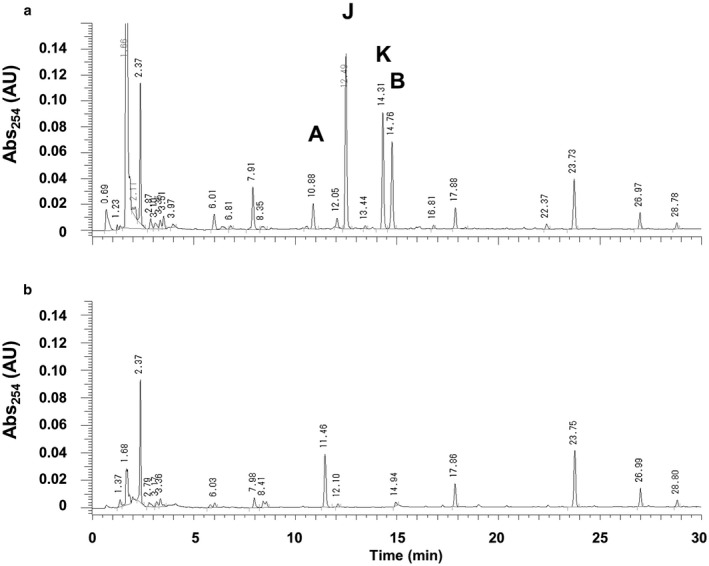
Influence of the *lecM* mutation on the production of ralfuranones by *Ralstonia solanacearum* strains. High‐performance liquid chromatography (HPLC) analysis of culture extracts from *R. solanacearum* strain OE1‐1 (a) and *lecM* mutant (OE1‐1‐*lecM*::EZ Tn*5*, b). The peaks of ralfuranones are marked as A, B, J and K.

To analyse the influence of LecM on *phc* QS‐positive regulation, we assessed the *phc* QS‐dependent production of ralfuranones by the *R. solanacearum* *lecM* mutant compared with strain OE1‐1. The *lecM* mutant produced less ralfuranones than OE1‐1 (Fig. 4).

We then analysed the *ralA *expression level in *R. solanacearum* strains grown on ¼ × M63 medium using qRT‐PCR assays. We observed a significantly lower *ralA* expression level in the *lecM* mutant than in the WT and *lecM*‐comp strains (*P* < 0.05, *t*‐test, Fig. 1).

### *lecM *mutant cells exhibit greater swimming motility than OE1‐1 cells

**Figure 5:**
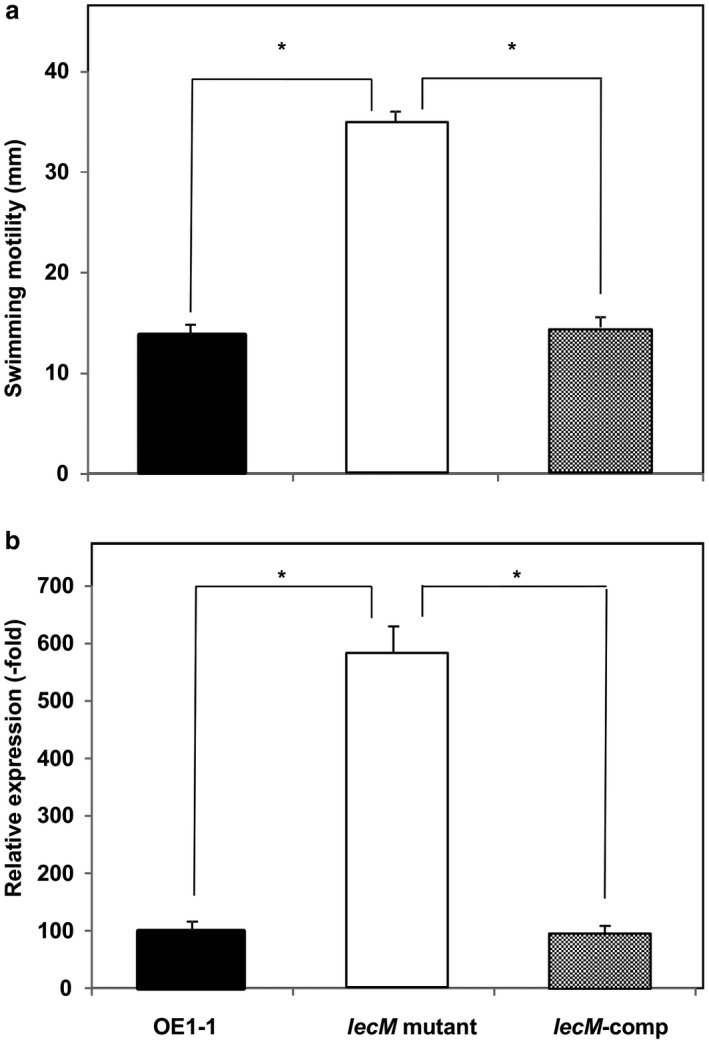
Influence of the *lecM* mutation on swimming motility (a) and *fliC* expression (b) of *Ralstonia solanacearum* strains. (a) *Ralstonia solanacearum* OE1‐1, *lecM* mutant (OE1‐1‐*lecM*::EZ Tn*5*) and native *lecM*‐expressing complemented *lecM* mutant (*lecM*‐comp) strains were grown on ¼ × M63 medium solidified with 0.25% agar. Values are presented as the means ± standard deviations of three replicates. The experiment was repeated three times, with five technical replicates in each experiment. Asterisks indicate values significantly different from those of WT strain OE1‐1 (*P* < 0.05, *t*‐test). (b) The *R. solanacearum* strains were grown in ¼ × M63 medium [to an optical density at 600 nm (OD_600_) = 0.3]. Total RNA was then extracted from the bacterial cells. The *rpoD* gene was used as an internal control for quantitative reverse transcription‐polymerase chain reaction. The gene expression levels are presented relative to the *rpoD* expression level. The experiment was conducted at least twice using independent samples, with similar results. Results for a single representative sample are provided. Values are presented as the means ± standard deviations of three replicates. Asterisks indicate values significantly different from those of OE1‐1 cells (*P* < 0.05, *t*‐test).

Flagella biogenesis is negatively regulated by *phc* QS, leading to the suppression of the swimming motility of *R. solanacearum* during *phc* QS activation (Tans‐Kersten *et al*., 2001)*.* To analyse the influence of LecM on *phc* QS‐negative regulation, we analysed the swimming motilities of the *R. solanacearum* strains. The *lecM* mutant exhibited a significantly greater swimming motility than WT strain OE1‐1 and the *lecM*‐comp mutant strain on ¼ × M63 medium solidified with 0.25% agar (*P* < 0.05, *t*‐test, Fig. 5a).

We analysed the expression level of *fliC*, encoding flagellin, in *R. solanacearum* strains grown on ¼ × M63 medium using qRT‐PCR assays. We observed significantly greater *fliC* expression levels in the *lecM* mutant than in the OE1‐1 strain and *lecM*‐comp strain (*P* < 0.05, *t*‐test, Fig. 5b).

### Expression analysis of *phc* QS‐related genes

We analysed the expression of the *phc* QS‐related genes *phcB* and *phcA* in *R. solanacearum* strains grown in ¼ × M63 medium (to OD_600_ = 0.3) using qRT‐PCR assays. There were no significant differences among the *lecM* mutant, *lecM*‐comp and OE1‐1 strains with regard to *phcB* and *phcA* expression levels (*P* > 0.05, *t*‐test, Fig. 1).

### The *lecM* mutation leads to a significantly reduced 3‐OH MAME content

**Figure 6:**
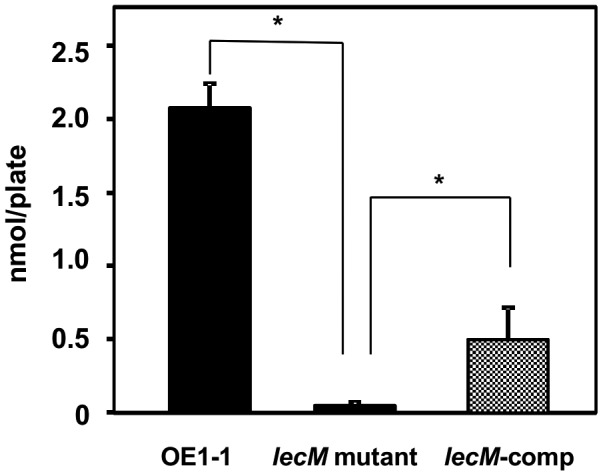
Influence of the *lecM* mutation on methyl 3‐hydroxymyristate (3‐OH MAME) purified from the *Ralstonia solanacearum *strains OE1‐1, *lecM* mutant (OE1‐1‐*lecM*::EZ Tn*5*) and native *lecM*‐expressing complemented *lecM* mutant (*lecM*‐comp). The experiment was conducted three times using independent samples. Asterisks indicate values significantly different from those of OE1‐1 (*P* < 0.05, *t*‐test).

The ralfuranone production‐deficient mutant (Δ*ralA*; Kai *et al*., 2014) exhibits a lower 3‐OH MAME content than the WT strain OE1‐1, although Δ*ralA* expresses *phcB* and *phcA*, similar to OE1‐1 (Mori *et al*., 2016), suggesting the involvement of ralfuranones in the 3‐OH MAME content. Because the *lecM* mutation leads to significantly reduced ralfuranone production, we determined the 3‐OH MAME contents of *R. solanacearum* strains. The *lecM* mutant exhibited a significantly lower 3‐OH MAME content than OE1‐1 (*P *< 0.05, Fig. 6). The *lecM*‐comp strain produced significantly more 3‐OH MAME than the *lecM* mutant (*P *< 0.05, *t*‐test, Fig. 6).

### Supplementation with 3‐OH MAME affects EPS I production of the *lecM* mutant

**Figure 7:**
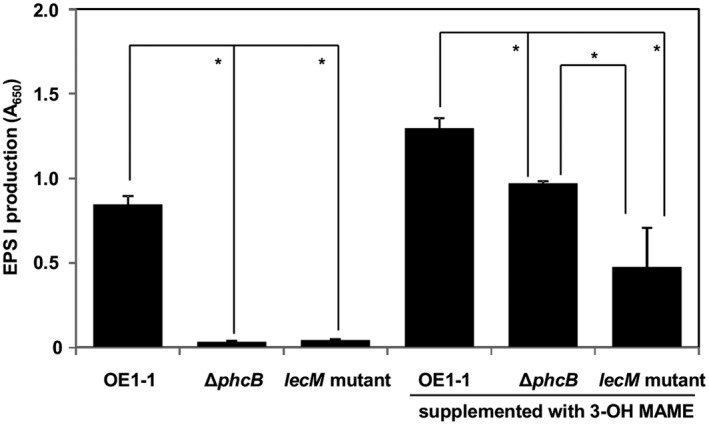
Production of exopolysaccharide I (EPS I) in *Ralstonia solanacearum* strains. Immunological quantification of EPS I in supernatants of *R. solanacearum* strains was performed using an enzyme‐linked immunosorbent assay with anti‐*R. solanacearum* EPS I antibodies. The *R. solanacearum* cells were also incubated in ¼ × M63 medium supplemented with methyl 3‐hydroxymyristate (3‐OH MAME) at concentrations of 1.0 µm. EPS I productivity was quantified by absorbance at 650 nm (A_650_). Bars indicate the standard errors. The experiment was repeated three times, with five technical replicates in each experiment. Asterisks indicate significant differences (*P* < 0.05, *t*‐test).

We analysed the *phc* QS‐dependent EPS I production of *R. solanacearum* strains. Similar to Δ*phcB*, the *lecM* mutant produced significantly reduced EPS I compared with strain OE1‐1 (*P* < 0.05, *t*‐test; Fig. 7). The application of 1.0 µm 3‐OH MAME significantly enhanced the EPS I production of the OE1‐1 strain (Table S4, see Supporting Information). The EPS I production of the Δ*phcB* strain increased significantly with the application of 1.0 µm 3‐OH MAME. However, the increased EPS I production by the *lecM* mutant supplemented with 1.0 µm 3‐OH MAME was significantly less than that in 3‐OH MAME‐supplemented OE1‐1 and Δ*phcB*.

### Invasion of the *lecM* mutant into xylem vessels

Previously, we have reported a loss in virulence for the *lecM* mutant when inoculated through roots by root dipping (Mori *et al*., 2016). Therefore, we analysed the infection of *R. solanacearum* strains into xylem vessels using the plate‐printing assay. The *lecM* mutant was detected beyond the inoculated roots and in both inoculated roots and stems of tomato plants, similar to OE1‐1 and* lecM*‐comp (Fig. S4, see Supporting Information).

## Discussion

The QS system in *R. solanacearum* consists of the *phc* regulatory elements, and PhcA functioning through the *phc* QS system plays a central role, leading to the virulence of this bacterial species (Genin and Denny, 2012). In strain OE1‐1, PhcB is a methyltransferase that synthesizes 3‐OH MAME (Kai *et al*., 2015). PhcS and PhcR constitute the two‐component regulatory system that corresponds to a threshold concentration of 3‐OH MAME, elevating the level of functional PhcA. Thus, the levels of functional PhcA are controlled by a conferment‐sensing system encoded by the *phcBSR* operon. However, it may be that, as in other bacteria, additional regulatory factors affect *phc* QS in *R. solanacearum *(Hikichi *et al*., 2017). The results of RNA‐seq analysis in this study suggested that positive regulation by PhcA may be independent of 3‐OH MAME production (Fig. 3; Table S1). Previously, we have reported the positive feedback regulation of *phc* QS by ralfuranones (Hikichi *et al*., 2017; Mori *et al*., 2018b). Furthermore, LecM is involved in the expression of genes regulated by *phc* QS (Fig. 3; Table S1). HrpG, a transcriptome regulator of the *hrp* regulon, positively regulates *lecM* expression, producing and extracellularly secreting LecM (Mori *et al*., 2016; Valls *et al*., 2006). LecM functions in both the attachment of bacteria to host plant cells and in bacterial cell–cell binding in biofilms (Meng *et al*., 2015). Furthermore, LecM is involved in the attachment of *R. solanacearum* cells to surfaces of host cells after invasion of intercellular spaces (Mori *et al*., 2016). This is followed by the construction of the type III secretion machinery and the secretion of effectors into host cells (Hikichi *et al*., 2017). OE1‐1 thus evades host innate immunity and grows vigorously on host cells, inducing *phc* QS. Functional PhcA also induces the production of LecM (Meng *et al*., 2015; Mori *et al*., 2016). Thus, LecM produced during *phc* QS is involved in the expression of genes regulated by *phc* QS.

Acyl homoserine lactones are a major class of autoinducer signal used by Gram‐negative proteobacteria for intraspecies QS (Ng and Bassler, 2009). LuxI and LuxR are essential for the QS control of bioluminescence in *Vibrio fischeri*. LuxI is the synthase in the QS signal *N*‐(3‐oxohexanoyl)homoserine lactone (Engebrecht and Silverman, 1984; Schaefer *et al*., 1996). LuxI catalyses the acylation and lactonization reactions between the substrates *S*‐adenosylmethionine and 3‐oxohexanoyl‐ACP (More *et al*., 1996; Schaefer *et al*., 1996). LuxR is the cytoplasmic receptor for the QS signal, as well as the transcriptional activator of the luciferase *luxICDABE *operon (Engebrecht and Silverman, 1984; Engebrecht *et al*., 1983). When the QS signal accumulates, it is bound by LuxR, and the LuxR–acyl homoserine lactone complex recognizes a consensus binding sequence upstream of the *luxICDABE *operon, activating its expression (Stevens *et al*., 1994). Because *luxI* expression is also activated by the QS signal‐bound LuxR, when the QS circuit engages, QS signal production is induced, and the surrounding environment is flooded with the signal molecule. Interestingly, lipolytic enzymes from culture‐based sources, capable of the hydrolysis of 3‐OH PAME, suppressed EPS production in *R.* *solanacearum* strain AW1‐3 (Shinohara *et al*., 2007). Furthermore, soil metagenome‐derived methyl ester hydrolases suppressed extracellular polysaccharide production in *R. solanacearum* strain GMI1000 (Lee *et al*., 2017). These results demonstrate that reduced content of extracellular 3‐OH MAME/3‐OH PAME results in reduced activity of *phc* QS. The *lecM* mutation led to lower 3‐OH MAME contents (Fig. 6), and the extracellular application of 3‐OH MAME induced *phc* QS‐dependent EPS I production by the *lecM* mutant to a lesser extent than the OE1‐1 and Δ*phcB* strains (Fig. 7). Interestingly, the *lecM* mutation did not affect *phcB* and *phcA* expression (Fig. 1). Thus, LecM may affect the activation of *phc *QS through the instability of extracellularly secreted 3‐OH MAME (Fig. S1).

PhcA functioning through *phc* QS‐induced expression of the ralfuranone production‐related genes *ralA* and *ralD* stimulated the production and extracellular secretion of ralfuranones (Kai *et al*., 2014; Schneider *et al*., 2009). Ralfuranones are involved in the positive feedback regulation of *phc* QS (Hikichi *et al*., 2017; Mori *et al*., 2018b). Because PhcA functioning through *phc* QS induces the production of LecM (Meng *et al*., 2015; Mori *et al*., 2016), this feedback regulation by ralfuranones leads to the induction of *lecM* expression during *phc* QS. Results in this study suggest that LecM may affect the activation of *phc *QS, involved in the regulation of *phc* QS‐dependent gene expression. Thus, the ‘autoinduction’‐positive feedback loops of *phc* QS mediated by ralfuranones and LecM in a population of OE1‐1 cells switched from a low‐cell‐density mode to a high‐cell‐density mode, leading to the induction of *phc* QS, enhancing *phc* QS (Fig. S1).

As in *phc* QS, both LecM and ralfuranones are required for OE1‐1 virulence (Hikichi *et al*., 2017; Kai *et al*., 2014; Mori *et al*., 2018a, 2018b). Thus, both the stability of 3‐OH MAME through LecM and the *phc* QS feedback regulated by ralfuranones may contribute to the full virulence of strain OE1‐1. Zuluaga *et al*. (2013) reported that the tomato apoplast is significantly richer in sugars, such as sucrose, fructose, glucose, galactose and mannose, than the xylem, and *R. solanacearum* probably catabolizes the abundant apoplast sugars. LecM exhibits mannose, fructose, fucose, galactose and arabinose affinity (Sudakevitz *et al*., 2004). Sudakevitz *et al*. (2004) reported that extracts of *R. solanacearum* strain GMI1000 had fructose/mannose‐specific haemagglutinating activities that could be attributed to LecM. Furthermore, OE1‐1 cells incubated in apoplast fluids produce mushroom‐type biofilms (Mori *et al*., 2016). On the contrary, OE1‐1 cells incubated in xylem fluids do not produce a mushroom‐type biofilm and aggregate formlessly. This suggests that intercellular spaces provide favourable conditions for mushroom‐type biofilm formation by OE1‐1 cells (Hikichi *et al*., 2017). Therefore, it is hypothesized that the LecM‐mediated regulation of strain OE1‐1, when invading intercellular spaces and xylem vessels, leads to mushroom‐type biofilm formation and formless cell aggregation, respectively, contributing to its virulence. Interestingly, when inoculated using root dipping, the *lecM* strain invaded xylem vessels (Fig. S3). However, the *lecM* mutant loses its virulence (Mori *et al*., 2016). Thus, LecM‐mediated regulation in OE1‐1 cells during mushroom‐type biofilm formation may be involved in the priming of OE1‐1 virulence after invasion of xylem vessels.

Ailloud *et al*. (2016) performed transcriptome analyses of *R. solanacearum* strains infecting host plants using RNA‐seq, and many virulence‐related genes were differentially expressed during infection, compared with *in vitro* growth, such as growth on a rich medium. A large majority of the PhcA controlled genes followed the same regulation pattern in both rich medium *in vitro* and after *in planta* growth, except for a set of HrpG‐HrpB regulated genes, including the type III secretion machinery and type III effectors, whose genes appeared to be specifically induced by PhcA in the plant environment, whereas this regulator repressed their expression in rich medium (Perrier *et al*., 2018). PhcA mediates a second strategic switch between the initial attachment of strain GMI1000 and its subsequent dispersal inside the host, and helps it to optimally invest resources and correctly sequence multiple steps in the bacterial wilt disease cycle (Khokhani *et al*., 2017). Our previous (Mori *et al*., 2016) and present studies on transcriptome analyses using RNA‐seq of *R. solanacearum* strains incubated in a poor medium, ¼ × M63, showed that the *phc* QS signalling pathway was regulated through LecM and ralfuranones in strain OE1‐1 (Figs 3 and S1). LecM‐ and ralfuranone‐mediated regulation was involved in mushroom‐type formation and invasion into xylem vessels, and contributed to full virulence. Strain OE1‐1 produces mushroom‐type biofilms when incubated in apoplast fluids from tomato plants, but not in xylem fluids (Mori *et al*., 2016). Therefore, there is a difference in the regulation of gene expression levels in *R. solanacearum* strains during early root colonization and in infected tomato xylem vessels. Taken together, further transcriptome analyses of *R. solanacearum* strains infecting host plants, focusing on LecM‐ and ralfuranone‐mediated regulation, will help to uncover the key regulatory mechanisms of *R. solanacearum *virulence.

## Experimental Procedures

### Bacterial strains, plasmids and growth conditions

We used the following *R. solanacearum* strains: OE1‐1 (Kanda *et al*., 2003), the *lecM* mutant (Mori *et al*., 2016), *lecM*‐comp (native *lecM*‐expressing complemented *lecM* mutant; Mori *et al*., 2016), Δ*phcB* (Kai *et al*., 2015), Δ*phcA* (Mori *et al*., 2016) and Δ*ralA* (Kai *et al*., 2014). The *R.* *solanacearum* strains were routinely grown in ¼ × M63 medium (Cohen and Rickenberg, 1956) at 30 °C. *Escherichia coli *strains were grown in Luria–Bertani medium (Hanahan, 1983) at 37 °C. Gentamycin (50 µg/mL) was used in selective media.

### Analysis of ralfuranones produced by *R. solanacearum* strains

*Ralstonia solanacearum* strains were grown in 100 mL of MGRLS medium (MGRL medium supplemented with 3% sucrose; Kai *et al*., 2014) in 300‐mL Erlenmeyer flasks at 30 °C with rotation (130 rpm) for 4 days. Following growth, the bacterial cultures were extracted three times with an equal volume of ethyl acetate. The combined extracts were dried over Na_2_SO_4_ and evaporated to dryness. The residues were dissolved in methanol (500 µL) and subjected to high‐performance liquid chromatography (HPLC) analysis: column, Inertsil ODS‐3 (250 mm × 4.6 mm, 5 µm; GL Sciences, Tokyo, Japan); column oven, 40 °C; flow rate, 1 mL/min; eluent, acetonitrile–H_2_O (20%–95% linear gradient in 40 min, then 95% acetonitrile for 10 min); injection volume, 10 µL. The experiment was conducted at least twice using independent samples, with similar results. Results for a single representative sample are provided.

### Analysis of 3‐OH MAME produced by *R. solanacearum* strains

*Ralstonia solanacearum* strains grown in B medium at 30 °C for 4–6 h were diluted to an OD_600_ of 1.0 with new medium. A sample (50 µL) of the cell suspension was pipetted onto a BG agar plate (90 mm, 25 mL; Kai *et al*., 2015), and the plate was incubated for 24 h at 30 °C. The BG agar was cut into small pieces and soaked in ethyl acetate (50 mL) for 2 h twice. The combined extracts were dried over Na_2_SO_4_ and concentrated. The residue was dissolved in ethyl acetate (2 mL) and subjected to gas chromatography‐mass spectrometry (GC‐MS) analysis. GC‐MS data were recorded with a GCMS‐QP2010 Plus (9, Kyoto, Japan) and an InertCap 5MS/NP column (25 m × 0.25 mm, 0.25 µm film, GL Sciences). The conditions used were as follows: injection, 1 mL (splitless; valve time, 60 s); injector temperature, 20 °C; carrier gas, He (at 0.8 mL/min); transfer line temperature, 300 °C; ion source temperature, 230 °C; electron energy, 70 eV. The temperature of the column oven was programmed as follows: 50 °C for 5 min, followed by an increase to 300 °C at 20 °C/min, with the temperature then being maintained at 300 °C for 5 min (Kai *et al*., 2015); data reflect three replicates each.

### RNA extraction, elimination of ribosomal RNA and sequencing

Two biologically independent experiments were conducted for each strain. Total RNA was isolated from *R. solanacearum* strains grown in ¼ × M63 medium (to OD_600_ = 0.3) using a High Pure RNA Isolation Kit (Roche Diagnostics, Mannheim, Germany). Ribosomal RNA was removed from the extracted total RNA using a Ribo‐Zero rRNA Removal Kit (Gram‐negative bacteria) (Illumina, Madison, WI, USA). Oriented paired‐end RNA‐seq (2 × 100 bp) was conducted by Hokkaido System Science (Sapporo, Japan) using an Illumina HiSeq 2000 system and the procedures recommended by Illumina. The adaptors and primers were designed by Hokkaido System Science. The selected inserts were 100 bp. We conducted the paired‐end sequencing of the libraries.

### Mapping and analysis of RNA‐seq data

Reads were trimmed using Cutadapt (version 1.1; https://code.google.com/p/cutadapt/) and Trimmomatic (version 0.32; https://www.usadellab.org/cms/?page=trimmomatic), and then mapped with TopHat (version 2.0.10; https://tophat.cbcb.umd.edu/). Read counts obtained for each of the samples are presented as FPKM, which was calculated with Cufflinks (version 2.2.1; https://cole-trapnell-lab.github.io/cufflinks/).

### qRT‐PCR

A 500‐ng total RNA template sample was reverse transcribed using a PrimeScript RT Reagent Kit (Takara, Otsu, Japan). A qRT‐PCR assay was conducted with a 20‐µL reaction mixture containing 1 µL cDNA stock and 10 pm primers (Table S5, see Supporting Information) using a SYBR GreenER qPCR Reagent System (Invitrogen, Tokyo, Japan). Reactions were completed in an Applied Biosystems 7300 Real‐time PCR system (Applied Biosystems, Foster City, CA, USA). The cycling parameters for all primers were as follows: 95 °C for 30 s; 40 cycles of 95 °C for 5 s and 60 °C for 31 s (Mori *et al*., 2016). Melting curve runs were completed at the end of each reaction to verify the specificity of the primers (i.e. presence of a single product). Relative quantification of gene expression was carried out according to the instructions for the Applied Biosystems 7300 Real‐time PCR system, using the comparative cycle threshold method for the calculation of the quantity value. All values were normalized against the *rpoD* expression level as an internal standard for each cDNA sample (Mori *et al*., 2016; Narusaka *et al*., 2011). There were no significant differences in the *rpoD* expression levels among *R. solanacearum* strains. The experiment was conducted at least twice using independent samples, with similar results. Results for a single representative sample are provided.

### Bacterial cell aggregation assay

The aggregation of *R. solanacearum *cells was measured *in vitro *using a slight modification of the polyvinylchloride microtitre plate assay described by O’Toole and Kolter (1998). Briefly, 5‐µL overnight cultures of *R. solanacearum *grown in ¼ × M63 medium adjusted to OD_600_ = 0.005 were used to inoculate 95 µL of ¼ × M63 medium in the wells of a polyvinylchloride microtitre plate (Nunc MicroWell plate; Thermo Fisher Scientific Inc., Waltham, MA, USA). Tomato apoplast fluid was added to the wells, and the plate was incubated at 30 °C for 24 h without shaking. To quantify the cell aggregation, 25 µL of 1.0% (w/v) crystal violet solution were added to the wells. After a 15‐min incubation, the unbound crystal violet stain was gently removed with a pipette, and the wells were washed with distilled water, 70% ethanol and distilled water again. The remaining crystal violet in each well was solubilized with 100 µL of 100% ethanol, and then quantified by measuring the absorbance at 550 nm. The resulting value was normalized according to the number of cells (OD_550_/OD_600_). This value was considered to represent the relative cell aggregation (Mori *et al*., 2016). The experiment was repeated three times, with seven technical replicates in each experiment.

### Swimming motility

Overnight cultures of *R. solanacearum *strains were washed with distilled water and diluted to a cell density of 1.0 × 10^8^ colony‐forming units (CFU)/mL. For the swimming assay, 3‐µL aliquots of cell suspensions were added to the centre of ¼ × M63 medium solidified with 0.25% agar. Motility was examined using three plates per strain. All plates were incubated at 30 °C. The diameters of the swimming areas were measured at 48 h post‐inoculation (Mori *et al*., 2018b). The experiment was repeated three times, with five technical replicates in each experiment.

### EPS I production

Quantitative analyses of EPS I production were conducted using an enzyme‐linked immunosorbent assay (Mori *et al*., 2016). The overnight culture of *R. solanacearum *strains was rinsed, and diluted to a cell density of 1.0 × 10^2^ CFU/mL. Then, 100 μL of these cell suspensions were spread on plates of ¼ × M63 agar medium and incubated for 2 days at 30 °C. Cells were resuspended to 1.0 × 10^5^ CFU/mL, and the cell density was confirmed through dilution plating. EPS I was quantified using anti‐*R. solanacearum *EPS I antibodies by an enzyme‐linked immunosorbent assay per 100 μL volume (1.0 × 10^4^ CFU) of cell suspension according to the manufacturer’s instructions (Agdia Inc., Elkhart, IN, USA). EPS I productivity was quantified by absorbance at 650 nm. EPS I production values were statistically analysed using the Tukey–Kramer honestly significant difference (HSD) test (*n* = 9) following an analysis of variance (ANOVA) with Easy R software (Saitama Medical Center, Jichi Medical University, Saitama, Japan; Kanda, 2013). The experiment was repeated three times, with five technical replicates in each experiment.

### Systemic infectivity assays

Tomato plants (*Solanum esculemtum* cv. Ohgata‐Fukuju) were grown in pots containing commercial soil (Tsuchitaro; Sumitomo Forestry Landscaping, Tokyo, Japan) in a growth room at 25 °C under 10 000 lx for 16 h per day, and watered with diluted 1/5 × Hoagland’s solution (Hikichi *et al*., 1999). The roots of 5‐week‐old tomato plants were soaked in bacterial suspensions at 1.0 × 10^8^ CFU/mL for 30 min and then washed in running water. Plants were then grown in water culture pots (Yamato Water Culture Pot No. 1, Yamato Plastic Co. Ltd., Yamatotakada, Japan) with fivefold‐diluted Hoagland’s solution. At 10 days post‐inoculation, after sterilization of plant surfaces with 70% ethanol, the roots and stems of five tomato plants were cut into three pieces each using razor blades (Kanda *et al*., 2008). The cut site of each piece (I, roots; II and III, stems; Fig. S4) was pressed onto strain‐specific media [Hara–Ono medium (Hara and Ono, 1983) for OE1‐1, Hara–Ono medium containing 50 µg/mL kanamycin for the *lecM* mutant and Hara–Ono medium containing 50 µg/mL kanamycin and 25 µg/mL gentamycin for *lecM*‐comp] and then incubated at 30 °C for 3 days.

## Conflicts of Interest

The authors declare that they have no conflicts of interest.

## Supporting information


**Fig. S1** ****Model of the regulation of *phc* quorum sensing (QS) mediated by RS‐IIL and ralfuranones in *Ralstonia solanacearum* strain OE1‐1.

**Fig. S2** ****Colonies of *Ralstonia solanacearum* strains OE1‐1, *lecM* mutant (OE1‐1‐*lecM*::EZ Tn*5*) and native *lecM*‐expressing complemented *lecM* mutant (*lecM*‐comp) on Hara–Ono medium (Hara and Ono, 1983).* Ralstonia solanacearum* strains were incubated on strain‐specific media (Hara–Ono medium for OE1‐1, Hara–Ono medium containing 50 µg/mL kanamycin for the *lecM* mutant and Hara–Ono medium containing 50 µg/mL kanamycin and 25 µg/mL gentamycin for *lecM*‐comp) at 30 °C for 2 days.

**Fig. S3** ****Correlations of gene expression level changes in *Ralstonia solanacearum* *phcB*‐deleted mutant (Δ*phcB*, a) and *phcA*‐deleted mutant (Δ*phcA*, b) with the expression level changes (i.e. ≥2 or ≤−2) of genes regulated by RS‐IIL encoded in *lecM*. The FPKM (fragments per kilobase of open reading frame per million fragments mapped) values for *R. solanacearum* strains OE1‐1, Δ*phcB*, Δ*phcA* and *lecM* mutant (OE1‐1‐*lecM*::EZ Tn*5*) were normalized prior to the analyses of differentially expressed genes.

**Fig. S4** ****Behaviour of *Ralstonia solanacearum* strain OE1‐1, *lecM* mutant (OE1‐1‐*lecM*::EZ Tn*5*) and native *lecM*‐expressing complemented *lecM* mutant (*lecM*‐comp) in tomato plants. *Ralstonia solanacearum* strains in tomato plants at 10 days after inoculation by root dipping were detected using the plate‐printing assay (Kanda *et al*., 2008). At 10 days post‐inoculation with *R. solanacearum* strains, the surfaces of the roots and stems were sterilized with 70% ethanol. The roots and stems of five tomato plants were then cut into three pieces each using razor blades. The cut site of each piece (I, roots; II and III, stems; Fig. 8b) was pressed onto strain‐specific media [Hara–Ono medium (Hara and Ono, 1983) for OE1‐1, Hara–Ono medium containing 50 µg/mL kanamycin for the *lecM* mutant and Hara–Ono medium containing 50 µg/mL kanamycin and 25 µg/mL gentamycin for *lecM*‐comp] and incubated at 30 °C for 3 days.

**Table S1** ****RNA sequencing data for all transcripts in *Ralstonia solanacearum* strains grown in ¼ × M63 medium.

**Table S2** ****(1) Predicted function of proteins encoded by positively RS‐IIL‐regulated genes among positively *phc* quorum sensing (QS)‐regulated genes in *Ralstonia solanacearum* strain OE1‐1 grown in ¼ × M63 medium. (2) Predicted function of proteins encoded by positively RS‐IIL‐regulated genes among positively *phc* QS‐regulated genes in *Ralstonia solanacearum* strain OE1‐1 grown in ¼ × M63 medium. (3) Predicted function of proteins encoded by positively RS‐IIL‐regulated genes among positively *phc* QS‐regulated genes in *Ralstonia solanacearum *strain OE1‐1 grown in ¼ × M63 medium. (4) Predicted function of proteins encoded by positively RS‐IIL‐regulated genes among positively *phc* QS‐regulated genes in *Ralstonia solanacearum* strain OE1‐1 grown in ¼ × M63 medium. (5) Predicted function of proteins encoded by positively RS‐IIL‐regulated genes among positively *phc* QS‐regulated genes in *Ralstonia solanacearum* strain OE1‐1 grown in ¼ × M63 medium.

**Table S3** ****Predicted function of proteins encoded by RS‐IIL‐negatively regulated genes among *phc* quorum sensing (QS)‐negatively regulated genes in *Ralstonia solanacearum* strain OE1‐1 grown in ¼ × M63 medium.

**Table S4** ****Tukey–Kramer analysis of exopolysaccharide EPS I production by *Ralstonia solanacearum* strains.

**Table S5** ****Primers used in this study.
