# Supplementary figures and images for: Contribution of a lectin, LecM, to the quorum sensing signalling pathway of Ralstonia solanacearum strain OE1‐1
Source: Mol Plant Pathol. 2018 Nov 6;20(3):334–45. doi: 10.1111/mpp.12757 (PMC6637872; doi:10.1111/mpp.12757)

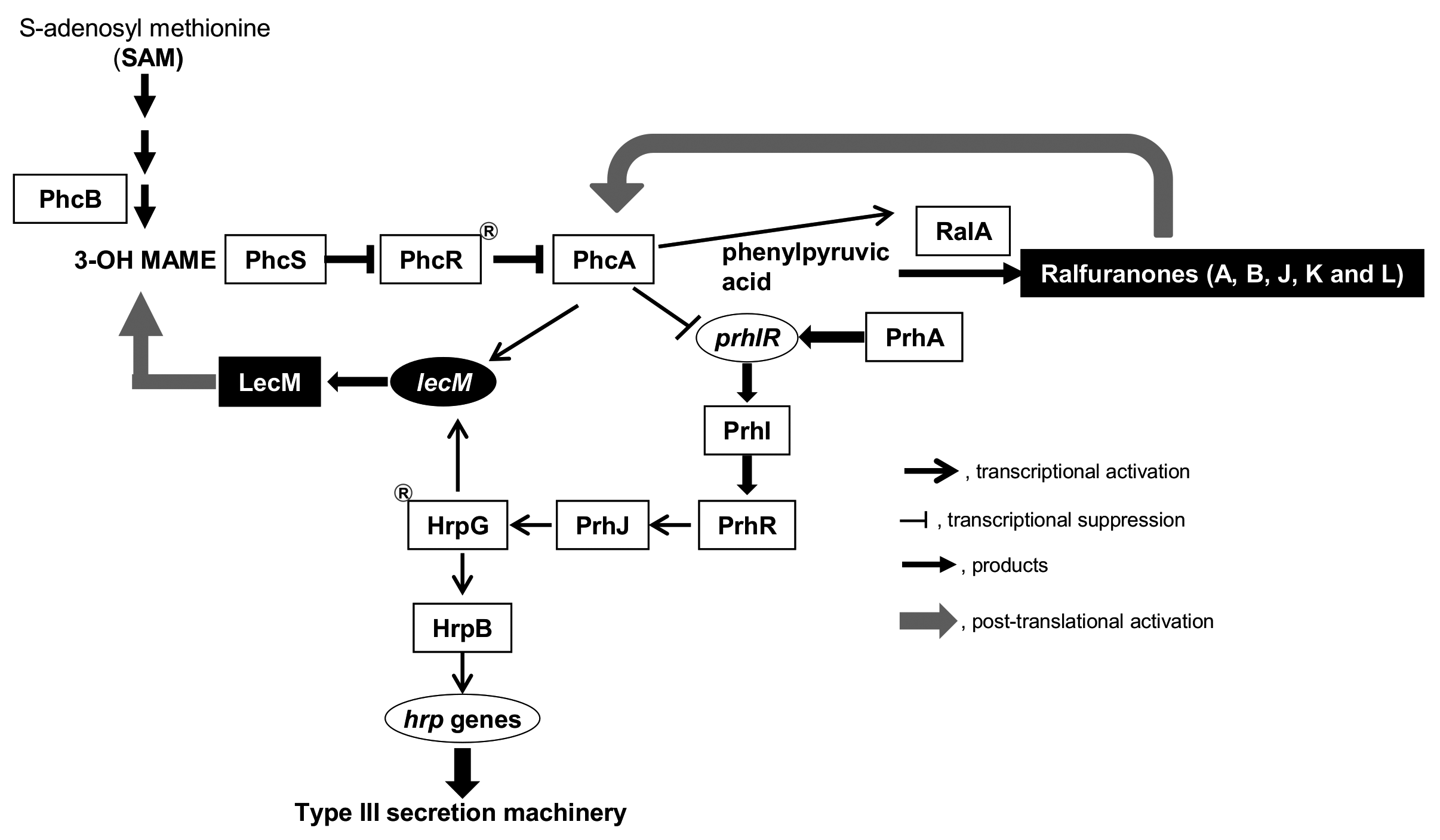

Supplement: Supplementary file 1 — Fig. S1 Model of the regulation of phc quorum sensing (QS) mediated by RS‐IIL and ralfuranones in Ralstonia solanacearum strain OE1‐1. [file MPP-20-334-s001.tif]

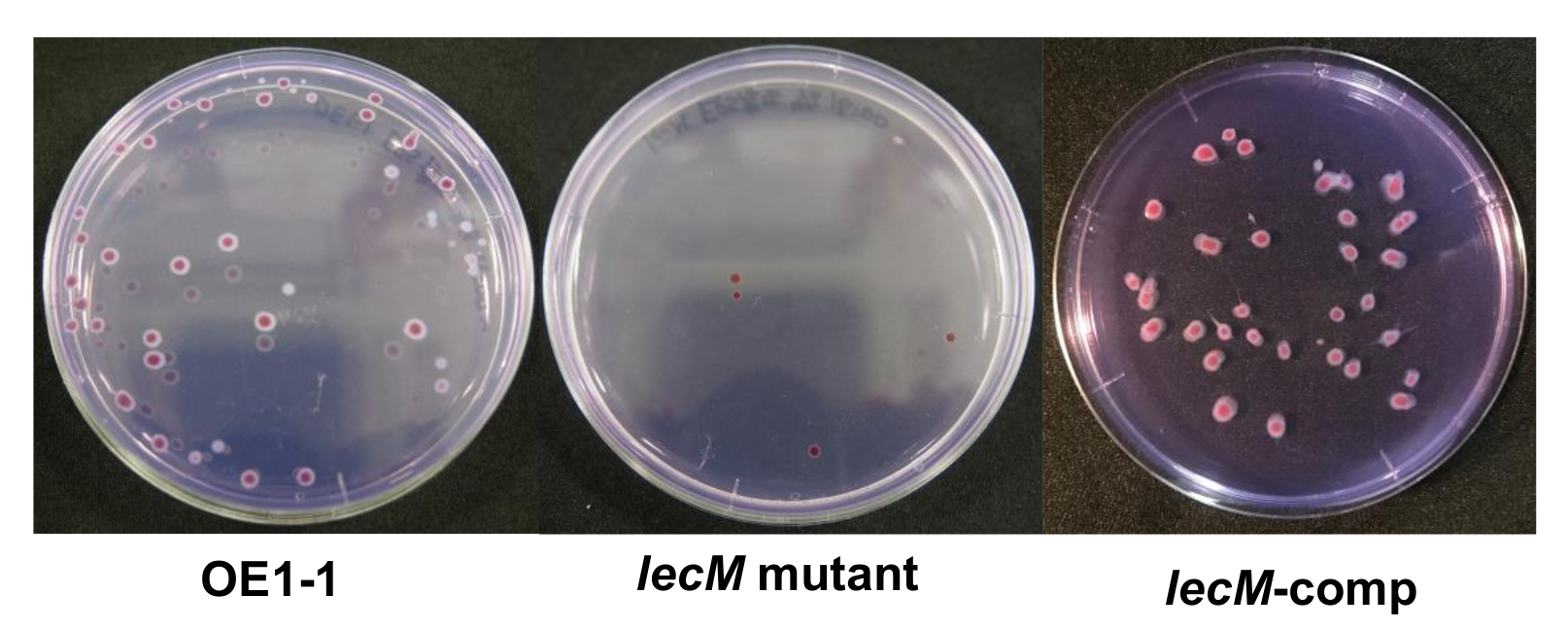

Supplement: Supplementary file 2 — Fig. S2 Colonies of Ralstonia solanacearum strains OE1‐1, lecM mutant (OE1‐1‐lecM::EZ Tn5) and native lecM‐expressing complemented lecM mutant (lecM‐comp) on Hara–Ono medium (Hara and Ono, 1983). Ralstonia solanacearum strains were incubated on strain‐specific media (Hara–Ono medium for OE1‐1, Hara–Ono medium containing 50 µg/mL kanamycin for the lecM mutant and Hara–Ono medium containing 50 µg/mL kanamycin and 25 µg/mL gentamycin for lecM‐comp) at 30 °C for 2 days. [file MPP-20-334-s002.tif]

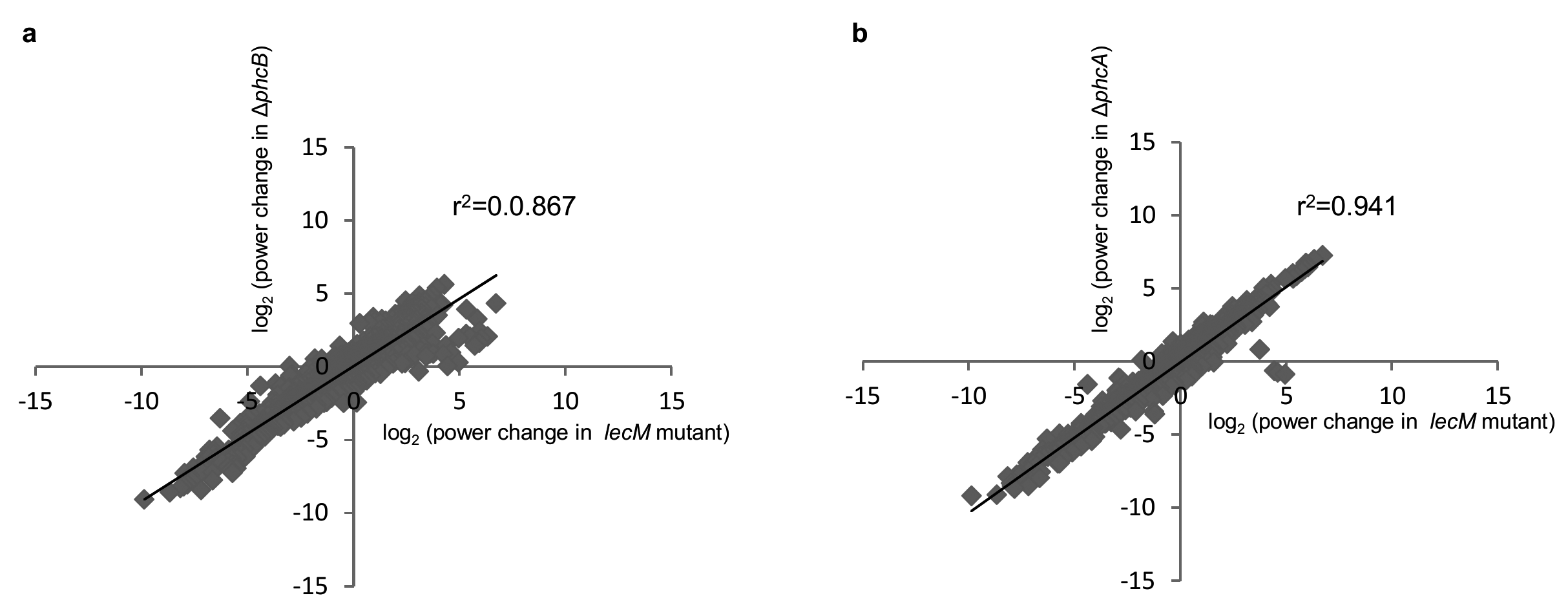

Supplement: Supplementary file 3 — Fig. S3 Correlations of gene expression level changes in Ralstonia solanacearum phcB‐deleted mutant (ΔphcB, a) and phcA‐deleted mutant (ΔphcA, b) with the expression level changes (i.e. ≥2 or ≤−2) of genes regulated by RS‐IIL encoded in lecM. The FPKM (fragments per kilobase of open reading frame per million fragments mapped) values for R. solanacearum strains OE1‐1, ΔphcB, ΔphcA and lecM mutant (OE1‐1‐lecM::EZ Tn5) were normalized prior to the analyses of differentially expressed genes. [file MPP-20-334-s003.tif]

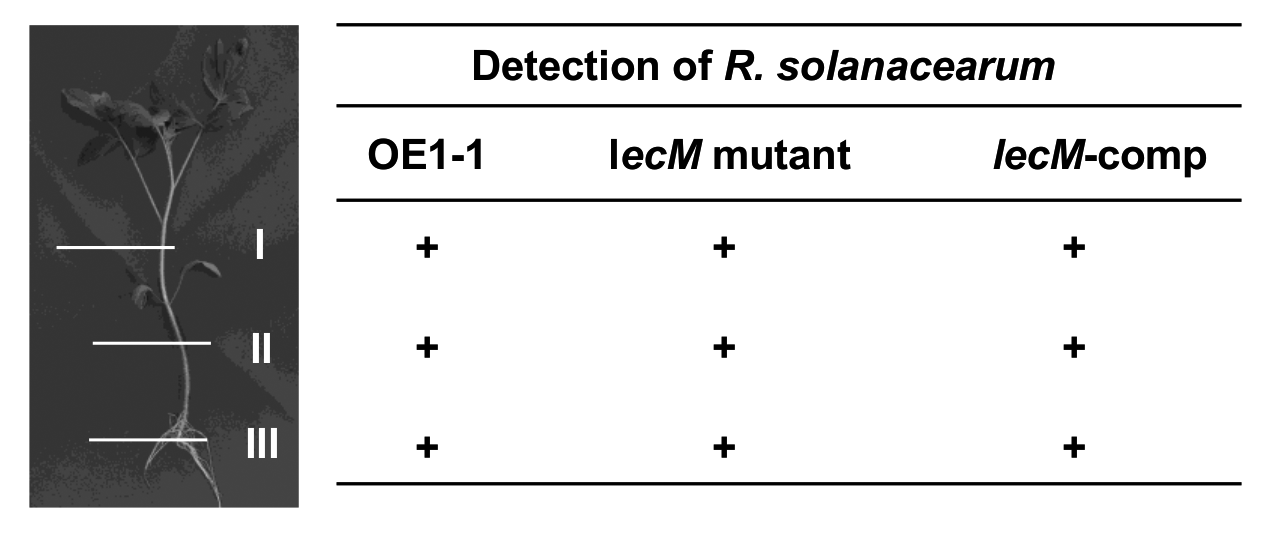

Supplement: Supplementary file 4 — Fig. S4 Behaviour of Ralstonia solanacearum strain OE1‐1, lecM mutant (OE1‐1‐lecM::EZ Tn5) and native lecM‐expressing complemented lecM mutant (lecM‐comp) in tomato plants. Ralstonia solanacearum strains in tomato plants at 10 days after inoculation by root dipping were detected using the plate‐printing assay (Kanda et al., 2008). At 10 days post‐inoculation with R. solanacearum strains, the surfaces of the roots and stems were sterilized with 70% ethanol. The roots and stems of five tomato plants were then cut into three pieces each using razor blades. The cut site of each piece (I, roots; II and III, stems; Fig. 8b) was pressed onto strain‐specific media [Hara–Ono medium (Hara and Ono, 1983) for OE1‐1, Hara–Ono medium containing 50 µg/mL kanamycin for the lecM mutant and Hara–Ono medium containing 50 µg/mL kanamycin and 25 µg/mL gentamycin for lecM‐comp] and incubated at 30 °C for 3 days. [file MPP-20-334-s004.tif]
