# Supplementary material for: Contribution of a lectin, LecM, to the quorum sensing signalling pathway of Ralstonia solanacearum strain OE1‐1
Source: Mol Plant Pathol. 2018 Nov 6;20(3):334–45. doi: 10.1111/mpp.12757 (PMC6637872; doi:10.1111/mpp.12757)
Supplement: Supplementary file 8 — Table S4 Tukey–Kramer analysis of exopolysaccharide EPS I production by Ralstonia solanacearum strains. [file MPP-20-334-s008.docx]

**Table S4.** Tukey–Kramer analysis on EPS I production by *Ralstonia solanacearum* strains

|  | EPS I production (A_650_) | | | |
| --- | --- | --- | --- | --- |
|  | diff | lwr | upr | *P* value |
| Δ*phcB*, OE1-1 | -0.8117500 | -0.940935143 | -0.6825649 | 0.0000000 |
| *lecM* mutant, OE1-1 | -0.8017500 | -0.930935143 | -0.6725649 | 0.0000000 |
| OE1-1 applied with 3-OH MAME, OE1-1 | 0.4515833 | 0.322398190 | 0.5807685 | 0.0000000 |
| Δ*phcB* applied with 3-OH MAME, OE1-1 | 0.1249167 | -0.004268476 | 0.2541018 | 0.0637643 |
| *lecM* mutant applied with 3-OH MAME, OE1-1 | -0.3692500 | -0.498435143 | -0.2400649 | 0.0000000 |
| *lecM* mutant, Δ*phcB* | 0.0100000 | -0.119185143 | 0.1391851 | 0.9999131 |
| OE1-1 applied with 1 μM 3-OH MAME, Δ*phcB* | 1.2633333 | 1.134148190 | 1.3925185 | 0.0000000 |
| Δ*phcB* applied with 1 μM 3-OH MAME, Δ*phcB* | 0.9366667 | 0.807481524 | 1.0658518 | 0.0000000 |
| *lecM* mutant applied with 1 μM 3-OH MAME, Δ*phcB* | 0.4425000 | 0.313314857 | 0.5716851 | 0.0000000 |
| OE1-1 applied with 3-OH MAME, *lecM* mutant | 1.2533333 | 1.124148190 | 1.3825185 | 0.0000000 |
| Δ*phcB* applied with 1 μM 3-OH MAME, *lecM* mutant | 0.9266667 | 0.797481524 | 1.0558518 | 0.0000000 |
| *lecM* mutant applied with 1 μM 3-OH MAME, *lecM* mutant | 0.4325000 | 0.303314857 | 0.5616851 | 0.0000000 |
| Δ*phcB* applied with 1 μM 3-OH MAME, OE1-1 applied with 1 μM 3-OH MAME | -0.3266667 | -0.455851810 | -0.1974815 | 0.0000000 |
| Δ*phcB* applied with 1 μM 3-OH MAME, OE1-1 applied with 1 μM 3-OH MAME | -0.8208333 | -0.950018476 | -0.6916482 | 0.0000000 |
| *lecM* mutant applied with 1 μM 3-OH MAME, Δ*phcB* applied with 1 μM 3-OH MAME | -0.4941667 | -0.623351810 | -0.3649815 | 0.0000000 |

Immunological quantification of EPS I in supernatants of *R. solanacearum* OE1-1, *phcB*-deleted mutant (Δ*phcB*) and *lecM* mutant (OE1-1-*lecM*::EZ Tn*5*) strains was performed using an enzyme-linked immunosorbent assay with anti-*R. solanacearum* EPS I antibodies. The *R. solanacearum* cells were also incubated in 1/4× M63 medium supplemented with methyl 3-hydroxymyristate (3-OH MAME) at concentrations of 1.0 µM.

Diff, differences in mean; lwr, a lower bound for the confidence interval of each contrast; upr, an upper bound for the confidence interval of each contrast.
