# Supplementary material for: Contribution of a lectin, LecM, to the quorum sensing signalling pathway of Ralstonia solanacearum strain OE1‐1
Source: Mol Plant Pathol. 2018 Nov 6;20(3):334–45. doi: 10.1111/mpp.12757 (PMC6637872; doi:10.1111/mpp.12757)
Supplement: Supplementary file 9 — Table S5 Primers used in this study. [file MPP-20-334-s009.docx]

**Table S5**. Primers used in this study.

| Name of Genes | Name of primers | Nucleotide sequences |
| --- | --- | --- |
| *phcB*  *phcA*  *epsB*  *fliC*  *rpoD* | phcB-FW3-514  phcB-RV3-1011  phcA-FW2  phcA-RV2  epsB-FW  epsB-RV2  fliC-FW2  fliC-RV2  rpoD-FW  rpoD-RV | 5ʹ-TACAAGATCAAGCACTACCTCGACTG-3ʹ  5ʹ-GTGCTGTACGCCATCCATCTC-3ʹ  5ʹ-TGATTCCGCTCGATTACGCG-3ʹ  5ʹ-CCTTTTCTGCATCGCCCTTAC-3ʹ  5ʹ-ATGGTCGAGCTGATGGATA-3ʹ  5ʹ-TGGAGCTGCTTGATCGTCTC-3ʹ  5ʹ -CAAACGCAAGGTATTCAGAACG-3ʹ  5ʹ -ATTGGAAGGTCGTCGAAGCCAC-3ʹ  5ʹ-ATCGTCGAGCGCAACATCCC-3ʹ  5ʹ-AGATGGGAGTCGTCGTCGTCGTG-3ʹ |
